# A brief guide to the science and art of writing manuscripts in biomedicine

**DOI:** 10.1186/s12967-020-02596-2

**Published:** 2020-11-10

**Authors:** Diego A. Forero, Sandra Lopez-Leon, George Perry

**Affiliations:** 1 Health and Sport Sciences Research Group, School of Health and Sport Sciences, Fundación Universitaria del Área Andina, Bogotá, Colombia; 2 MSc Program in Epidemiology, School of Health and Sport Sciences, Fundación Universitaria del Área Andina, Bogotá, Colombia; 3 Global Drug Development, Novartis Pharmaceuticals Corporation, East Hanover, NJ USA; 4 Department of Biology and Neurosciences Institute, The University of Texas at San Antonio, San Antonio, TX USA

**Keywords:** Peer review, Abstracting and indexing, Publications, Writing, Biological science disciplines

## Abstract

Publishing articles in international scientific journals is the primary method for the communication of validated research findings and ideas. Journal articles are commonly used as a major input for evaluations of researchers and institutions. Few articles have been published previously about the different aspects needed for writing high-quality articles. In this manuscript, we provide an updated and brief guide for the multiple dimensions needed for writing manuscripts in the health and biological sciences, from current, international and interdisciplinary perspectives and from our expertise as authors, peer reviewers and editors. We provide key suggestions for writing major sections of the manuscript (e.g. title, abstract, introduction, methods, results and discussion), for submitting the manuscript and bring an overview of the peer review process and  of the post-publication impact of the articles.

## Introduction

Publishing articles in international scientific journals is the current primary approach for the communication of validated research findings and ideas. Scientific papers are commonly used as a major input for evaluations of researchers and institutions [1, 2]. However, taking into account the evolving and multidimensional landscape of the publishing process, there is a need for additional updated training in the science and art of writing manuscripts for scientific journals.

Few articles have been published previously about the different aspects needed for writing high-quality articles [3–6]. In this article, we provide an updated and brief guide for the multiple dimensions needed for writing manuscripts in the health and biological sciences, from current, international and interdisciplinary perspectives and from our expertise as authors, peer reviewers and editors, extending and complementing previous publications about this topic. The writing of manuscripts in biomedicine has its own standards, including the availability of multiple guidelines for reporting different types of studies, which are discussed in this article.

## General recommendations

One of the first steps before starting to write an article should be to read the main papers that have been previously published on the subject. The first search might be focused on the available literature reviews and meta-analyses, and key for a scientist, the technique of performing a proper literature review [7]. Science advances by building on what it is known and there is no point in re-inventing the wheel [8].

It has been suggested, when writing scientific papers, to keep it short, compact and simple, avoiding the excessive use of adjectives and adverbs [9]. If you read a word or sentence and it does not add anything, delete it.

The success of an article depends on the quality of primary data and their analyses, on the way it is written and on the clearness of the tables and figures. It is fundamental to follow the current standards of research integrity (such as avoiding plagiarism and data manipulation) [10]. Both negative and positive results should be published, to avoid publication bias [11].

Authors should keep in mind that scientific writing is a process that involves multiple steps, takes time, dedication and inspiration, and involves patience, motivation, analytical thinking and adherence to high-quality standards [86]. Table 1 provides an important number of online resources that facilitate the writing of scientific manuscripts.

**Table 1:** Key digital resources to facilitate the writing of articles in the health and biological sciences

Online Resource	Website	Use
JANE	jane.biosemantics.org	To identify journals and authors with similar articles
Journal Suggester (Taylor and Francis)	authorservices.taylorandfrancis.com/journal-suggester	To identify candidate journals
Journal Finder (Elsevier)	journalfinder.elsevier.com	To identify candidate journals
Journal Suggester (Springer Nature)	journalsuggester.springer.com	To identify candidate journals
Journal Finder (Wiley)	journalfinder.wiley.com	To identify candidate journals
Scimago Journal Rank	scimagojr.com	To identify ranking of journals
NLM Catalog	ncbi.nlm.nih.gov/nlmcatalog	To identify indexing of journals
Journal Citation Reports*	jcr.clarivate.com	To identify IF of journals
Directory of Open Access Journals	doaj.org	To identify OA journals
ORCID	orcid.org	IDs for researchers
Publons	publons.com	Information about peer reviewers
Open Science Framework	osf.io	Data repository
GitHub	github.com	Code repository
figshare	figshare.com	Data repository
Google Scholar	scholar.google.com	Search of citations
Mendeley	mendeley.com	Management of references
Zotero	zotero.org	Management of references
EndNote*	endnote.com	Management of references
bioRxiv	biorxiv.org	Preprints repository
medRxiv	medrxiv.org	Preprints repository
ICMJE recommendations	icmje.org/recommendations/browse/	International criteria for writing manuscripts
MeSH on Demand	meshb.nlm.nih.gov/MeSHonDemand	Selection of MeSH
Equator Network	equator-network.org	Guidelines for reporting
Altmetric	altmetric.com	It provides alternative metrics
PubPeer	pubpeer.com	To comment on published articles
Retraction Watch	retractionwatch.com	It provides information about retracted articles

*IF* Impact Factor; *OA* Open Access; *MeSH* Medical Subject Headings

^*^Commercially available

## Authors

Following international recommendations for the authorship of articles in the biomedical sciences, such as the ones from the International Committee of Medical Journal Editors (ICMJE), is a fundamental topic in scientific publications, in order to avoid ghost and gift authorship practices [12, 13]. In general, authors should have a significant involvement in these 4 points: (1) study concept/design, data collection or data analysis/interpretation (2) drafting/revising the manuscript, (3) approving the final version and (4) holding responsibility for accuracy and integrity of all aspects of the reported research [14].

There is a trend for the increase of the number of authors over the years [15], which is a reflection of globalization and the increasing complexity of medical research [16]. In the last two decades, there has been an increased use of consortia authorship with very long lists of authors, usually derived from international mega-collaborations. Authors from non-English speaking countries might have to take into account the current standards for names (two first names and one last name), to avoid confusion in the indexing processes in databases. Authors with two last names can hyphen their two last names to avoid confusing their first last name with a middle name, although the use of ORCID identifiers facilitates the disambiguation of author profiles.

The meaning of the order of the listed authors varies between fields. In many disciplines, the author order indicates the magnitude of the contribution, with the last author usually representing the principal investigator [17]. It is possible to have an equal co-authorship, either for the first or corresponding authors [18].

## Title and abstract

The Title [19] and the Abstract [20] are the two most visible items of the article [21], as they are the main sections indexed in bibliographic databases. These two elements compete for the reader’s attention; therefore both should be informative, accurate, attractive, concise, clear and specific [19, 20]. It is advisable that the title of the manuscript reflects the actual findings of the work and be concise.

The Abstract section should provide a brief description of the main sections of the manuscript, describing key methods, findings and conclusions. It is recommended that the abstract be specific, clear, unbiased, honest, concise, precise, stand-alone, complete, and scholarly [22]. An important number of medical journals ask for structured abstracts. Usually, keywords are provided at the end of the Abstract section and the use of Medical Subject Headings (MeSH) as keywords is quite helpful.

## Introduction section

Although the standards of the length of the Introduction vary between scientific fields (for example, they are longer in psychology journals), it is recommended that the introduction section should be concise, avoiding long reviews about the topics of the article. It has been proposed that the introduction section be designed as a cone or funnel, starting with the main points of the general topic, followed by a highlight of the existing knowledge gap, the hypothesis or main question of the article and ending with a brief overview of the approach of the current work [23].

Another recommendation is to keep it simple, including three main paragraphs: the first paragraph explaining what is known, the second what is not known and the third what the objective of the study is and explain what it will add to the scientific knowledge. When stating what is known, it should not be a full review of the literature, but it should be the essential information needed to understand the background. Information from the introduction should not overlap with the discussion. The paragraph explaining what is unknown should be focused on helping the reader understand why the research is being performed. The last paragraph should state the research question or hypothesis [24]. It is important to cite key articles (both recent reviews and related primary works) and to highlight the novelty of the current work.

## Methods section

This section is essential and should be written to facilitate other researchers enabling them to replicate the study. This section has been compared to a recipe, which includes all the ingredients and how they need to be combined [25].

Key details of methods employed, such as overall design of the study, inclusion and exclusion criteria, sample sizes and statistical power, should be described [26]. Another way to subdivide it is with subheadings that might include: study design, setting, subjects, data collection and data analysis [25]. The incorporation of data about the origins of samples and validated criteria for diagnoses is indispensable, including key references to validated instruments and methodologies. Description of approval by institutional ethics committees and use of informed consent, when needed, is fundamental. In the case of the use of equipment and reagents, details of the respective manufacturers are needed. Statistical and bioinformatic analyses should be described clearly, including the details of statistical tests and the software used [27–30]. It is fundamental that all the results described in the Results section correlate with the procedures described in the Methods section.

## Results section

The Results section should provide an adequate and complete description of the main findings of the work carried out. It is suggested to avoid the repetition of the same exact content of the Tables or Figures and to leave the interpretation of the results of the findings to the Discussion section [31]. The main messages and details of the Results section should be provided in the Figures and Tables. No interpretation should be provided in this section.

The results section should be seen as a mirror of the methods: for every method provided, there should be a corresponding result. Subheadings can be included and some suggestions might be: recruitment/response, characteristics of the sample, findings from primary analyses, secondary analyses and additional findings [32]. Exact *p* values should be presented and must always be shown together with the estimates and confidence intervals. There should be a consistency with the number of decimal places presented in the results section and in the tables. It is common to present one or two decimals places. Always present the absolute number of cases, in addition to relative measures (e.g. percentage was 22% -33/150-) [32].

## Tables and figures

Tables facilitate the detailed presentation of the results and they should be constructed adequately. Abbreviations are useful for avoiding repetitions of phrases and should be explained in the footnotes [33]. Each table or figure should be self-explanatory, and there should be no need to read the text to be able to understand it. They have to be presented in the same chronological order, following how they are presented in the text [34].

For tables where a lot of information is presented, the *p* values that are statistically significant can be presented in bold. In case of long or complex tables, it is helpful to provide them as supplementary files, leaving the key data in the tables of the main text. It is important to provide details of statistical significance in the table, in order to avoid going back and forth between the tables and the text to read key data.

The creation of figures for scientific articles involves data visualization. A major element in the creation of figures is their focus on the representation of key findings without biases, avoiding the generation of overly complex figures. In addition, it is important to remove the repetition of the same data that is also presented as tables in the main manuscript. Description of key conventions should be provided in detail in the figure legends and it is important to avoid the misrepresentation of data [35], particularly digital enhancement. As the large majority of journals are published and distributed in digital formats, there are no actual restrictions for the adequate use of colors in scientific images. In case of photographs, it is important to follow the guidelines of the journals regarding image size and resolution. In addition, other recommendations are related to the use of adequate tools and parameters for the generation of figures [36].

## Discussion section

**Fig. 1:**
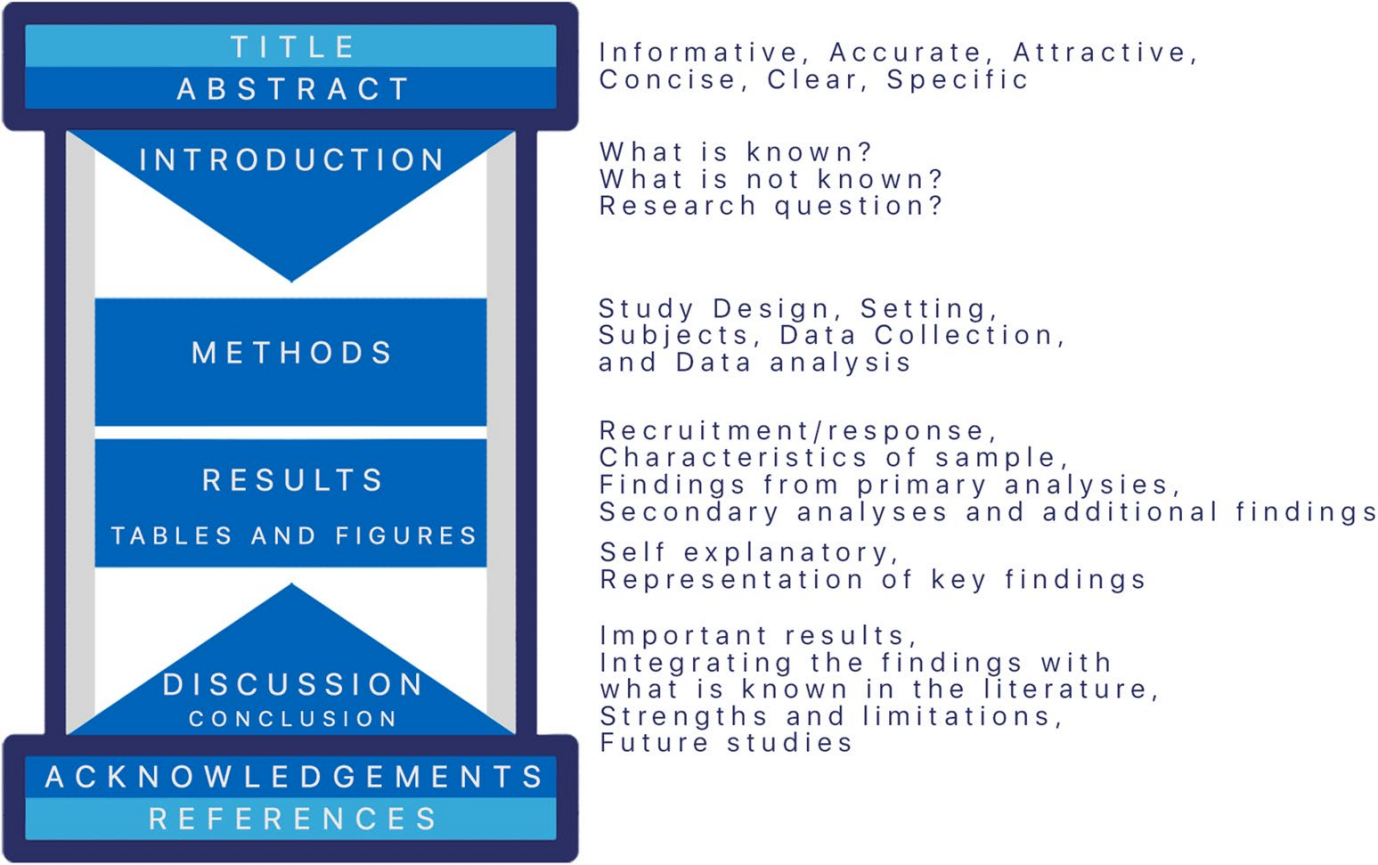
A graphical overview of the general structure of research articles

It has been proposed that the general outline of the discussion can be seen as an inverted funnel. Thus, it has been suggested that the configurations of the introduction and discussion sections can have, together, the form of an hourglass [37] (Fig. 1). The first paragraph is usually a summary of the important results, focused on answering the research question. The next paragraphs should focus on integrating the findings with what is known in the literature. If there are different findings, each should have a separate paragraph. The discussion of each result should follow the same order of the methods and results. A balanced contextualization of findings of the current study should be provided by citing the key previous original articles and related reviews that put the results in perspective [38]. If there are differences between the findings and previously published studies, the differences and similarities of the results and studies should be stated.

It is important to list the strengths and the limitations of the study. An explanation of the implications of those limitations should be included. An essential point is to include the needs and the perspectives for future studies. It can be stated that the results need replication or to highlight new questions that appeared after the analyses. This point can be of great guidance for future studies and can help the advance of science. It is highly advisable to avoid very long discussion sections and overstatements about the actual findings. The discussion section should not have results that were not described in the Results section. The last paragraph should include a conclusion that clearly states what the study adds to the knowledge.

## References section

Although each journal usually has its own citation style, the Vancouver style is quite common in medical journals. There are several freely and commercially available programs (such as EndNote, Zotero or Mendeley) that facilitate the citations process and the generation of the bibliography, including the details for multiple citation styles. They can help to organize, store, download -and most importantly- format the references to the style requirements of the journal you want to submit to. By having the references in these programs, it is easy to reformat the style for any other journal in a matter of seconds.

Always try to cite the original source behind a key statement, making sure that the reference you mention is not only mentioning another source. If you need to choose among several references, take into consideration the level of evidence, the year of publication and the quality of the work [39].

It is important to verify that the bibliography includes all the publications cited and to check issues with names of authors or journals. Several journals have limitations in the number of citations for certain types of publications.

## Acknowledgments and other sections

Usually, the authors thank their funding agencies for their economical support for the studies carried out. In addition, it is possible to include acknowledgements to people who helped with the development of the work (technical support, for example) or in the writing of the manuscript (such as corrections of use of the English language) [40]. In several cases, the journals ask for declarations about ethical considerations and declarations of the roles of individual authors (such as the design of the study and/or the collection or analysis of the data) [41]. Declarations of potential conflicts of interest is fundamental for the transparency of scientific activities [12, 42].

## Supplementary data

With modern high-throughput methods, the size of the analyzed datasets is becoming larger and larger. This means that there is a growing need to provide access to the large datasets as supplementary files (such as spreadsheets or pdf files) or to include them in publicly available repositories (such as OSF or figshare) [43]. In addition, certain fields have specific guidelines asking authors to submit their data to specific online repositories (such as the NCBI GEO database for whole genome expression data) [44].

## Review articles and other types of publications

There are two main types of review articles: systematic reviews and narrative reviews. In the case of systematic reviews and meta-analyses there are important standards to follow, including the need for well-defined search strategies [45]. For the writing of narrative reviews [46, 47], it is essential to define its scope and current needs and it is highly advisable to construct tables and figures to consolidate and visualize the key information. Articles for case reports follow a different structure and there are recommendations about their development [48].

## Reporting guidelines

It is important to follow published guidelines for the reporting of studies in clinical research, such as STROBE for observational studies [49], STROBE-ME for molecular epidemiology studies [50], STREGA for genetic association studies [51], PRISMA for systematic reviews and meta-analyses [52], TRIPOD for prediction models of diagnosis or prognosis [53], CONSORT for clinical trials [54], CARE for case reports [55] and AGREE II for practice guidelines [56], in addition to ARRIVE 2.0 for animal research [57]. For molecular and cellular analyses, there are several important guidelines, such as MIQE for qPCR [58, 59], flow cytometry [60], cell death [61], mutational analyses [62], simulation experiments [63] and gene nomenclatures [64, 65].

## Find the best candidate journals

There are several aspects that the authors should take into account in the selection of a journal, such as local standards of publications, the visibility or impact of the journals and their affinities with the topics of the manuscripts. It is highly advisable to verify the indexing of the journals in key databases, such as PubMed, Scopus/Scimago (quartiles) and Journal Citation Reports (impact factor) [66, 67]. Finally, authors should be careful with the growing number of predatory journals [68], which commonly mention spurious impact factors [69]. Another way to determine which journal is suitable is to see the list of the references in your study. Before selecting the journal, read all the instructions and make sure the scope of the journal and editor preference fits your manuscript. Make a list of 3 to 5 journals, and rank them [70]. In several cases, sending a pre-submission enquiry to the editor of the journal is helpful [71]. There is a growing trend for the initial divulgation of manuscripts as preprints, in repositories such as bioRxiv and medRxiv [72].

## Submission and peer review

It is fundamental to follow the guidelines for authors of the selected journal. In addition to manuscript files, tables, figures and supplementary data, it is common that the authors provide a cover letter (highlighting the main contributions of the work) in their submissions. In the cover letter it is recommended to include: (1) Your request to submit your work (mentioning the title). (2) 2–3 sentences summarizing the significance of the work (importance, main finding, message) (3) A statement of the relevance to the journal audience (eg. A related work published in the journal) (4) Any statement required from the journal, such as that the material has not been submitted/published elsewhere [73].

There are differences in peer review practices between journals. In many cases, there are two or more peer reviewers in a single-blind approach (the authors do not know the identities of the reviewers). In other cases, there is an approach based in double-blind, in which the reviewers also do not know the identities of the authors. In recent years, there has been an increase in the implementation of open peer review, in which the identities and concepts of the reviewers are publicly available.

## Answer to peer reviewers

When addressing the comments and questions of the peer reviewers do it in a new document. Copy/paste all comments and number them. For each comment briefly respond and indicate where the change was made in the manuscript. The response should be in present tense or past present (e.g. We now present; we have added to the first paragraph).

Make the changes in the paper with “track changes” or highlighting the change in another color. Be thankful and respectful to each reviewer and editor and take each comment very seriously. If you disagree with the comment, add solid evidence, adding references or key data [74].

The process of providing adequate answers to peer reviewers and editors and of the incorporation of their suggestions into the revised manuscript is an important challenge [75] in the publishing of an article.

## Open science

**Fig. 2:**
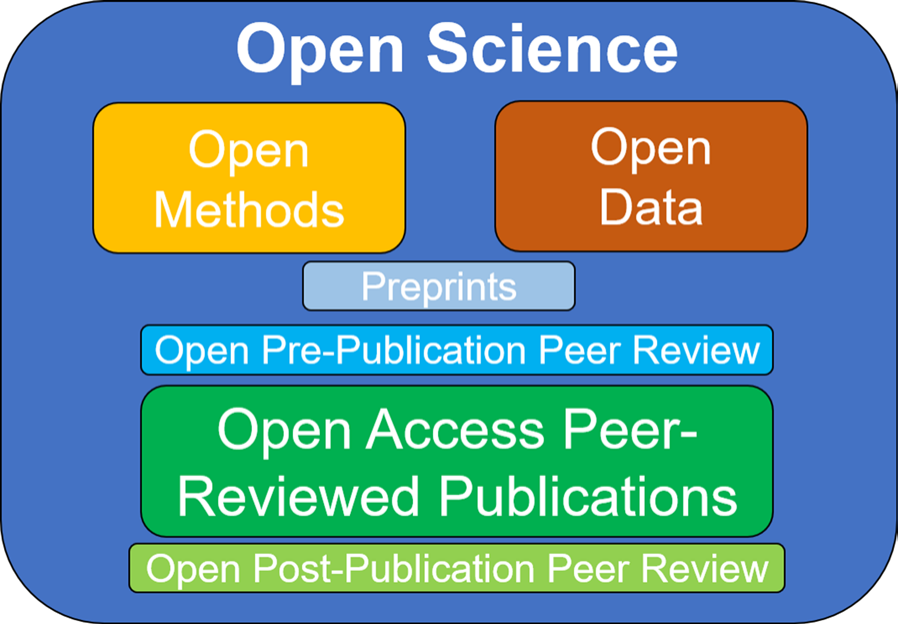
An overview of the different dimensions and components of Open Science

Interest in Open Science practices has been growing in recent years, considering their advantages to facilitate the access to information and their potential to increase the reproducibility and the quality of research findings [76–80]. It has been shown that open access articles [81] have advantages in terms of the amount of citations [82] and that articles that provide links to repositories with primary data have also have a higher citation count [43]. Open Science, in addition to open peer review, also involves open protocols, materials [8, 83] and data (Fig. 2).

## Post-publication impact

Citation counts are one of the main ways to measure the scientific impact of publications, allowing the development of multiple metrics, such as the H index [84], to measure the influence and visibility of scientists and research groups [1]. Recently, there is a growing use of alternative metrics [85], which measure other types of article mentions (such as social networks, blogs and news, recorded by Altmetric) or downloads. There are platforms (such as PubPeer and Retraction Watch) that allow comments on published articles, facilitating divulgation of possible issues on reported findings (among others) and to visualize information about retracted articles.

## Conclusions

The quality of scientific publications is directly related to the careful revision by peer reviewers of the manuscript, in order to improve the submitted manuscript. This process means that receiving feedback is a constant process and that authors should have the resilience to receive rejections and recommendations for major changes [2]. In addition, authors can have feedback from collaborators before submitting the manuscript (including revision of the use of the English language) and they can benefit themselves from the experience of being peer reviewers [86]. In the current scientific environment, publishing an article is not the end of a process; it is the beginning: the article is beginning its journey of being read and analyzed by people around the world.

The writing of a scientific article is a work of art that is honed with experience. The more publications you have, the easier it is to write a manuscript. The collaboration between authors can be very enriching and give rise to new projects and new learnings. The contribution to science and to following generations comes with every single article one publishes. Therefore, one should always strive for the best.

## Data Availability

Not applicable.
